# Permeability Characteristics of a New Antifungal Topical Amphotericin B Formulation with γ-Cyclodextrins

**DOI:** 10.3390/molecules23123349

**Published:** 2018-12-18

**Authors:** Carmen López-Castillo, Carmina Rodríguez-Fernández, Manuel Córdoba, Juan J. Torrado

**Affiliations:** 1 Pharmaceutics and Food Technology, School of Pharmacy, Complutense University, 28040 Madrid, Spain; carlopezcastillo@gmail.com; 2 Microbiology and Parasitology, School of Pharmacy, Complutense University, 28040 Madrid, Spain; carmina@ucm.es

**Keywords:** amphotericin, γ-cyclodextrin, rheology, permeability, antifungal, topical

## Abstract

Amphotericin B is a low soluble broad-spectrum antifungal agent. Cyclodextrins can be added to amphotericin formulations to enhance both their solubility and antifungal properties. Semisolid amphotericin formulations containing gamma cyclodextrin (AGCD) were prepared and compared with two reference formulations—one of them without any solubility enhancer (A) and the other with DMSO (ADMSO). Rheological, the permeability through hairless mouse skin and antifungal characteristics of the different formulations were evaluated. All three semisolid formulations show low thixotropy characteristics. ADMSO was the formulation with the least consistency, lowest viscosity, and greatest extensibility. The AGCD formulation had the opposite behavior and had both the greatest consistency and viscosity and the lowest extensibility. The lowest permeability was obtained with the reference A formulation while both AGCD and ADMSO had a similar permeability enhancement. According to the antimicrobial in vitro efficacy trials, the AGCD formulation showed 45–60% more activity than the reference A formulation. It can be concluded that γ-cyclodextrin is a useful excipient to improve the solubility, permeability, and antifungal activity of amphotericin B in semisolid topical formulations.

## 1. Introduction

**Figure 1:**
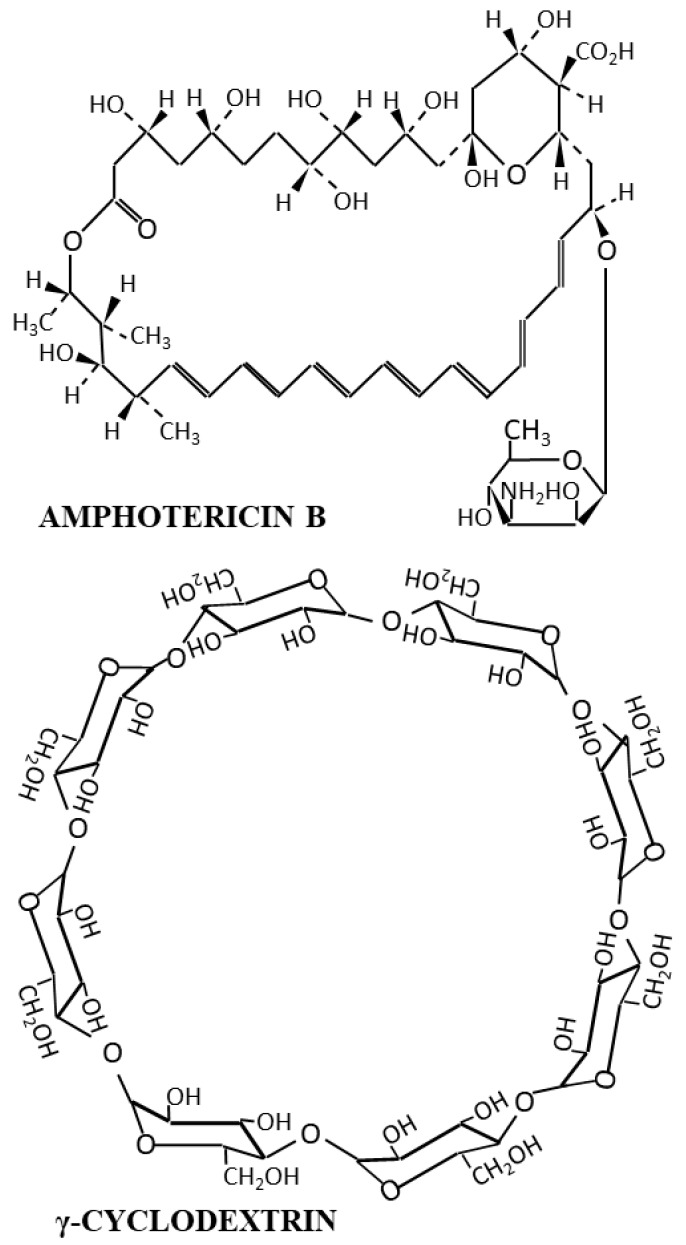
Chemical structure of AmB and γ-cyclodextrin.

Amphotericin (AmB) is a polyene broad-spectrum antifungal agent with uncommon incidence of primary resistances [1]. The low solubility of AmB is one of the most important limitations to developing new formulations [2]. In order to increase the low AmB solubility, different approaches have been applied, such as solubilization with polysorbate 80 [3] or interactions with hydrophilic molecules. Some of the hydrophilic agents that have been combined with AmB are related to polyethylene glycol [4], chitosan [5,6,7], or cyclodextrins [8,9,10]. Particularly, the effect of γ-cyclodextrin on the AmB aggregation state and the consequences on both the activity and toxicity have been previously described [11,12,13,14]. Moreover, the combination of γ-cyclodextrin with AmB has a synergic effect on the antifungal activity of ophthalmic eye drops [15] and topical gel formulations [16]. Several pathogens, such as *Candida albicans,* and mycobacteria enter host cells via a cholesterol-enriched membrane lipid raft microdomains, which can be altered by two different and synergistic mechanisms [17,18]. One of them is related to the depletion of the host membrane cholesterol by the cyclodextrins. The other one is the cholesterol and ergosterol complexation with AmB, resulting in a disruption of the membrane cells. These two mechanisms explain the synergistic antifungal activity of the cyclodextrin and AmB combination. Figure 1 shows the chemical structure of AmB and γ-cyclodextrin. Although the antifungal activity of the topical AmB and γ-cyclodextrins formulations have been previously reported [15,16], the permeability characteristics through the skin of hydrophobic formulations containing AmB solubilized with cyclodextrins (AGCD) has not been described yet, and this is the main aim of this work. Reference formulations without cyclodextrins (A) and with DMSO (ADMSO) were also studied. Moreover, the rheological and antifungal characteristics were also evaluated.

## 2. Results

### 2.1. Rheological Behavior

**Figure 2:**
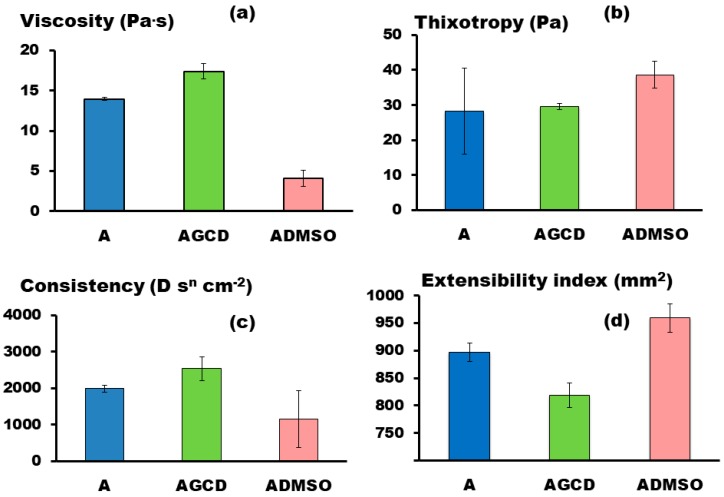
Mean results ± 95% confidence intervals corresponding to the rheological behavor of the three semisolid AmB tested formulations: (**a**) mean viscosity (Pa·s) at 15 rpm (significant differences, *p* < 0.01); (**b**) thixotropy (Pa) (no significant differences, *p* > 0.1); (**c**) consistency (D s^n^ cm^−2^) (significant differences, *p* < 0.01); (**d**) extensibility index (mm^2^) (significant differences, *p* < 0.05). Key: (A) semisolid AmB simple reference formulation, (ADMSO) is an AmB reference formulation with DMSO and (AGCD) is an AmB formulation with γ-cyclodextrins. Statistics was performed with an analysis of variance (ANOVA) test (n = 3 samples for each formulation).

Figure 2 shows the viscosity (2a), thixotropy (2b), consistency (2c), and extensibility index (2d) of the three semisolid AmB formulations that were tested. The rheological behavior depends on the different composition of the formulations, and it was pseudoplastic for all of them. Logically, the incorporation of a liquid solvent, such as DMSO, in the ADMSO formulation decreases the viscosity and consistency, while it increases its extensibility index. On the other hand, the incorporation of cyclodextrins, which have cohesive characteristics, increases the viscosity and consistency, while decreases its extensibility.

### 2.2. Permeability

**Figure 3:**
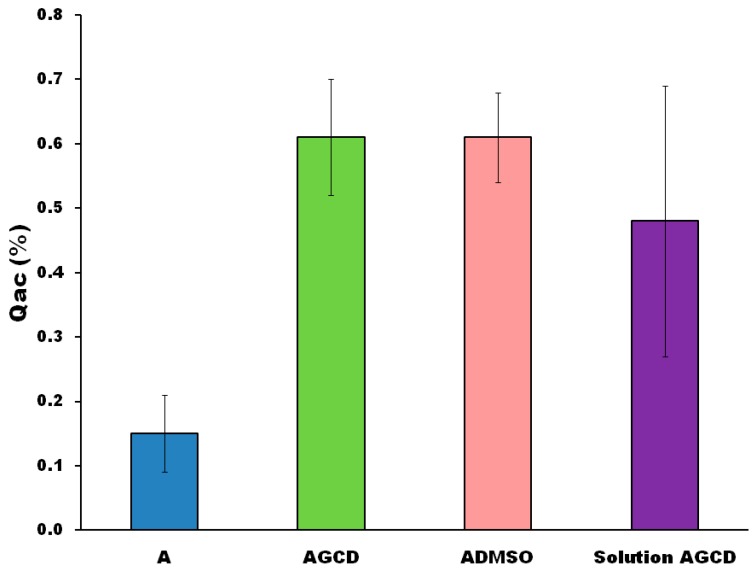
Average results ± 95% confidence intervals of AmB accumulation at the skin (Qac) obtained with three semisolid tested formulations and a liquid solution of AmB dissolved with cyclodextrins. Key: (A) semisolid AmB simple reference formulation, (ADMSO) is a semisolid AmB reference formulation with DMSO, (AGCD) is a semisolid AmB formulation with γ-cyclodextrins. A liquid solution of AmB solubilized with γ-cyclodextrin is also tested as reference. Statistics data treatment was performed with an ANOVA test (n = 9 for each formulations). The AmB skin accumulation obtained with formulation A was significantly (*p* < 0.01) lower than the skin accumulations obtained with the others formulations.

Figure 3 shows the mean results of the permeability, expressed as AmB accumulated at the skin. The semisolid AGCD and ADMSO formulations show a significant (*p* < 0.01) accumulation of AmB on the skin when compared with the reference semisolid formulation. Moreover, the local permeability of a liquid solution of AmB prepared by dissolving the AmB with the γ-cyclodextrins (Solution A) also results in an accumulation on the skin that is significantly (*p* < 0.01) higher than the reference semisolid A formulation. The main difference among these four formulations is that in formulation A, the AmB is neither non-solubilized nor formulated with an excipient to enhance its solubilization. No significant differences (*p* > 0.01) were observed between the two semisolid AGCD and ADMSO formulations. Therefore, the permeability effect of γ-cyclodextrin is similar to DMSO. Interestingly, in our experimental conditions, none of the formulations were able to promote AmB diffusion through all of the skin layers, because AmB was not detected in the liquid of the receptor chamber. This is important, as it could mean that non-systemic effects should be expected with these formulations.

### 2.3. Antifungal In Vitro Activity

**Figure 4:**
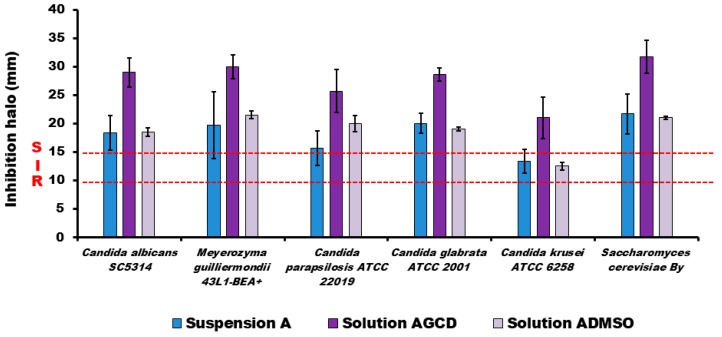
Antifungal in vitro activity of three liquid AmB formulations against different species of *Candida* and *Saccharomyces cerevisiae* (*Saccharomyces cerevisiae By*). Disks were embedded with 20 µL of AmB reference suspension (Suspension A), an AmB solution with cyclodextrins (Solution AGCD), or an AmB solution with DMSO. The growth inhibition halo diameters were measured at points where there was a complete inhibition of yeast growth. The isolates were classified as susceptible (S) to AmB when the inhibition zone was ≥15 mm, resistant (R) when it was ≤10 mm, and intermediate (I) or susceptible-dose dependent when the inhibition zone was between 10–14 mm [19]. Growth inhibition halo diameters are expressed as mean ± standard deviation (SD) are in mm. Each experiment was performed in triplicate. No antifungal activity, measured as halo inhibition (<1 mm), was observed with Orabase^®^, DMSO, and γ-cyclodextrins products.

Figure 4 shows the antifungal in vitro activity of the three liquid AmB tested formulations. The antifungal activity of the AmB solution containing cyclodextrins was always significantly (*p* < 0.01) higher than the other tested liquid formulations.

**Figure 5:**
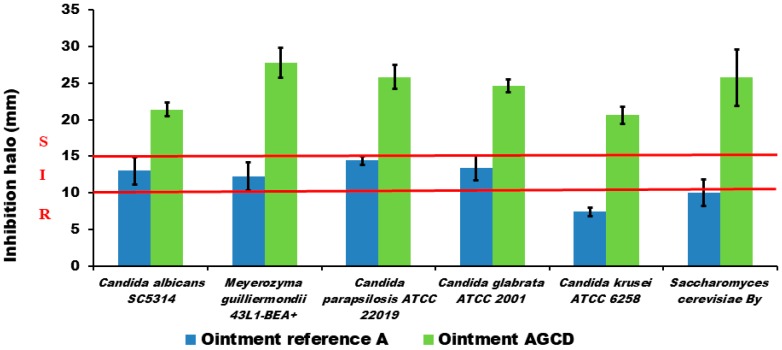
Antifungal in vitro activity of two semisolid AmB formulations against different species of *Candida* and *Saccharomyces cerevisiae* (*By*). One is an AmB reference ointment, and the other is an ointment with the AmB previously solubilized by cyclodextrins (Solution AGCD). The growth inhibition halo diameters were measured at points where there was a complete inhibition of yeast growth. The isolates were classified as susceptible (S) to AmB when the inhibition zone was ≥15 mm, resistant (R) when it was ≤10 mm, and intermediate (I) or susceptible-dose dependent when the inhibition zone was between 10–14 mm [19]. Growth inhibition halo diameters are expressed as mean ± SD and are in mm. Each experiment was performed in triplicate. No antifungal activity measured as halo inhibition (<1 mm) was observed with Orabase^®^, DMSO, and γ-cyclodextrins products.

Figure 5 shows the antifungal activity of a reference AmB ointment (A) and the ointment containing AmB previously solubilized by cyclodextrins (AGCD). The AmB formulated with cyclodextrins significantly (*p* < 0.01) improves the antifungal activity of the ointment.

## 3. Discussion

One important factor related to the formation of a true inclusion complex between poor soluble drugs, such as AmB and cyclodextrins, depends on the drug molecular size and the hydrophobic cavity of the cyclodextrins. γ-cyclodextrins have been reported as being more appropriate for AmB complexing than α- and β-cyclodextrins [8]. The AmB solubility enhancement is linearly related to the γ-cyclodextrin concentration, and it can be increased by up to 200-fold. Ruiz et al. [16] have characterized the AmB as follows: cyclodextrin interaction as a 1:1 molar ratio although only about one out of every 64 γ-cyclodextrin molecules interacts with AmB to form the water-soluble complex. The γ-cyclodextrin ratio of interaction and the differences in the molecular weights justifies the weight ratio of 1:100 reported by Ruiz et al. [16], which is also used in this work. A more complete description of the AmB complex characteristics, including stability of the complexes, has been previously reported [14,15,16]. In previous papers, it was proven that the solubilization of AmB by γ-cyclodextrins was useful to prepare hydrophilic ophthalmic [15] and gel topical [16] formulations. However, most topical marketed antifungal formulations in Spain are creams [20]. The inclusion of hydrophobic components in the formulation can have an occlusive effect on the skin, and modifies the permeability characteristics of the active component. Moreover, the antifungal enhancer effect obtained by the combination of cyclodextrins with AmB could be altered in a less hydrophilic medium. In order to test the permeability and efficacy of the AmB solubilized by γ-cyclodextrins, an ointment formulation based on the available marketed excipient Orabase^®^ was developed. This excipient is a hydrophobic base prepared for pharmaceutical compounding. Its composition [21] includes mineral oil at a concentration between 80–90% (CAS: 8042-47-5), astragalus gummifer gum (2–7%) (CAS: 9000-65-1), pectin (2–7%) (CAS: 9000-69-5), polyethylene (2–7%) (CAS: 9002-88-4), and chitosan (1–5%) (CAS: 9012-76-4). The composition of this base is especially useful to fix and maintain the drug at the oral cavity for up to 2 h. Moreover, the bioadhesion of the product can be also helpful for coating catheters so as to prevent nosocomial infections.

Figure 3 shows that the results of the accumulation of AmB at the skin achieved with the ointment containing AmB solubilized by cyclodextrins is not statistically (analysis of variance (ANOVA), *p* > 0.01) different from either the reference semisolid formulations containing DMSO, or the AmB solution. All of these three formulations have a significantly (ANOVA, *p* < 0.01) higher skin accumulation than the reference semisolid AmB formulation, named as formulation A. Therefore, the skin accumulation of AmB is related to the solubilization effect obtained by either the solvent DMSO or the cyclodextrins. Furthermore, the permeability enhancement can be also related, not only to the effect on the solubilization, but also on a possible disruption of the skin structure. The use of DMSO as a penetration enhancer is well known and well documented, but the skin structural disruptions are also indicators of local toxicity and irritability. In this sense, the topical irritability of DMSO has been reported by many authors, although it is also true that DMSO shows less toxicity than other penetration enhancers like *N*-methyl-2-pyrrolidone, Azone, oleic acid, methyl laurate, or benzyl alcohol [22]. In contrast to chemical penetration enhancers like DMSO, cyclodextrins are safe for topical application, and are also used for several formulations intended for ocular absorption. So, if the AmB permeability enhancement is similar, the incorporation of cyclodextrins as a topical excipient should be preferable to DMSO. In our experimental conditions, no effect of the consistency of formulation on AmB skin accumulation was detected, because not significant (*p* > 0.01) differences were observed between the semisolid ointments and the liquid AmB solution. It is important to note that, although the γ-cyclodextrin formulations were able to increase the AmB skin accumulation, in none of the tested formulations was it observed that AmB could passed through the skin barrier to the receptor chamber. This is an indicator of the low systemic toxicity expected with these new formulations. According to the results of the ex vivo permeability study, cyclodextrins can increase external AmB skin accumulation where the infection is usually located, without significant passage to the deepest skin layers.

Finally, the antifungal in vitro activity was tested both in liquid and in semisolid AmB formulations. The antifungal enhancer effect of the incorporation of cyclodextrins to the AmB formulation was observed with all of the tested strains, as it was previously reported [15,16]. In future works, it could be interesting to relate the increase in activity with the composition of the fungal membrane structure. It is important to compare the permeability and activity results obtained with the ointments with DMSO and γ-cyclodextrins. The permeability results show a similar skin accumulation for the ointments with DMSO than with γ-cyclodextrins. Nevertheless, the antifungal activity (see Figure 4) results reported for the formulation with γ-cyclodextrins was clearly higher than for the ointment with DMSO. In our opinion, this difference between the permeability/antifungal behavior between both formulations is related to the synergic antifungal action of the combination of γ-cyclodextrins with AmB. The hydrophobic ointment (AGCD) containing AmB solubilized with cyclodextrins is a promising topical antifungal product formulation.

## 4. Materials and Methods 

### 4.1. Materials

Amphotericin B USP quality was purchased to Azelis (Barcelona, Spain), Orabase^®^ (Fagron Ibérica, ref. 31188, Tarrasa, Spain), γ-cyclodextrin (Ashland Industries Europe Pharma, Cavamax^®^ W8 ref. 826761, Schaffhausen, Switzerland), and dimethyl sulfoxide (Scharlau, ref. SU01512500, Sentmenat, Spain). All of these products were of Pharmacopoeia quality. 

### 4.2. Formulations

Table 1 shows the composition of the three semisolid AmB formulations elaborated. The simple A reference formulation was elaborated by mixing the AmB with Orabase^®^ in a morter. In the ADMSO formulation, the first step was to dissolve AmB in DMSO, and afterwards, the resulting solution was mixed with Orabase^®^ in a morter. The elaboration of the AGCD started by dissolving 0.125 g of AmB in a purified water solution containing 12.5% (*w*/*w*) of dissolved γ-cyclodextrin at basic 12 pH (NaOH), according to the method previously reported by Ruiz et al., 2014 [16]. Once the AmB was dissolved, phosphoric acid was added to adjust the pH to 5.5. This solution (Solution ADMSO) was frozen and freeze-dried. The semisolid AGCD formulation was elaborated by mixing the freeze-dried powder containing AmB and γ-cyclodextrin with Orabase^®^ in a mortar. 

Table 2 shows the composition of the three liquid AmB formulations, elaborated in a similar way to the semisolid formulations previously described.

### 4.3. Rheological Studies

Thixotropy (Pa) is a reversible time-dependent decrease in the viscosity of the formulation at a particular shear rate, while consistency (D s^n^ cm^−2^) is relative to the viscosity of the formulation when it is exposed to a shear stress. The consistency was determined according to the Herschel Bulkley model for a pseudoplastic product, as the antilogarithm of the slope of the log–log relationship between shear stress and shear rate. Both the thixotropy and consistency characteristics were evaluated with the data obtained in the rheological studies. The rheological studies were evaluated at 25 ± 0.1 °C in a previously calibrated Brookfield rotational rheometer (model HB, Brookfield Engineering Laboratories, Stoughton, MA, USA) with a cone-plate geometry (spindle CP52). The data were collected using the Rheocalc 32 software (Brookfield Engineering Laboratories, Stoughton, MA, USA). The apparent viscosity (Pa·s) and shear stress (D·cm^−2^) were determined over a speed rate from 0 to 30 rpm and a shear rate from 0 to 30 s^−1^, increasing and decreasing sequentially in order to obtain the complete upward and downward rheogram for each formulation. The changes in the semi-solid structure were adjusted to the Power Law model for a pseudoplastic behavior, according to the following expression: SS = m·SR^n^, where SS is the shear stress, SR is the shear rate (s^−1^), m is the consistency coefficient calculated as the antilogarithm of the slope of the log–log relationship between shear stress and shear rate, and n is the flow behavior index or Power Law index (being 1 in Newtonian fluids, and lower than 1 in the pseudoplastic systems). The thixotropy (Pa) was evaluated through hysteresis (area between the ascending and descending curves), where a greater area meant a greater thixotropic effect, as an indicator of the capacity of the system to reestablish the micro-structural changes that takes place when exposed to shaking along a certain period of time.

For the extensibility test, the surface area of a semisolid formulation depends on the weight applied over the formulation. The methodology has been previously reported [23]. Approximately 1 g of the formulation is deposited on the surface of a methacrilate plate, then another plate is placed over the formulation, and finally, different weights of 50, 100, and 200 g are deposited. The surface of the formulation gives an idea of the mean extensibility index (mm^2^).

### 4.4. Permeability

Dorsal mouse skin was removed from immunocompetent female hairless mice SKH1-HrBR (Charles River Laboratories, L’Arbresles, France) of 6 weeks of age weighing approximately 25 g, that had been previously euthanized according to the Ethical Committee Regulations of the Complutense University of Madrid. The experimental design and housing conditions were approved by the Committee of Animal Experimentation of the university and the regional authorities (Community of Madrid) (ES280050001165). The skin was washed with purified water and then the hypodermis tissue was manually removed. The skin was mounted between the donor and receptor chamber of a flow through a diffusion cell (PermeGear^®^ ILC-07, Riegelsville, PA, USA). The effective diffusion area was 0.785 cm^2^ and the donor and receptor cell volumes were of 1.1 and 0.85 cm^3^, respectively. The phosphate buffer solution (pH 7.4) was maintained at 37 ± 0.5 °C and the fluid in the receptor chambers was continuously stirred. Accurately weighed (100 mg) prepared AmB formulations were spread in a thin layer on the skin membrane in the donor chambers and kept for 24 h. The flow rate at the receptor cell was 1.5 mL/24 h. Each formulation was tested 12 times and the following three samples were obtained from each experiment: (i) remnant sample in the donor chamber, (ii) skin membrane, and (iii) sample in the receptor chamber. The remnant sample in the donor chamber was removed from the surface of the skin membrane after the experiment, and was diluted with DMSO, centrifuged, diluted, filtered, and assayed by HPLC. Once washed, the skin membrane was dried, frozen if required, moltured, and the possible AmB remnant was extracted through successive mixing of the skin with a mixture of methanol, as follows: purified water (50:50), diluted with acetonitrile, stirred in a vortex, centrifuged, filtered, and assayed by HPLC. The liquid of the receptor chamber was stirred in a vortex, centrifuged, filtered, and assayed by HPLC. The AmB concentration was assayed by a validated HPLC method previously described [24]. Briefly, modular HPLC (Jasco, Tokyo, Japan) equipment was used. The flow rate was 1 mL/min of a mobile phase containing a mixture of acetonitrile as follows: acetic acid: purified water at proportions: 52:4.3:43.7. The stationary phase was a BDS Hypersil^®^ C18 column (Thermo Scientific, Bellefonte, PA, USA) 5 µm, 250 × 4.6 mm. The injection volume was 100 µL and the detection wavelength was 406 nm. The samples were filtered through Millex HV PVDF 0.45 µm filters (Millipore^®^, Carrigtwohill, Ireland).

### 4.5. Antifungal In Vitro Activity

Antifungal activity against five different species of Candida: *C. albicans* SC5314 ATCC MYA2876, *C. guilliermondii* (*Meyerozyma guilliermondii*) 43L1-BEA+, *C. parapsilosis* ATCC 22019, *C. glabrata* ATCC 2001, *C. krusei* ATCC 6258, and *Sacharomyces cerevisiae By*4741 (MA Ta his3∆1 leu2 ∆0 met15∆0 ura3∆0) was tested. All of the yeast strains were kindly provided by the Department of Mycrobiology and Parasitology, Faculty of Pharmacy, Complutense University of Madrid (Madrid, Spain).

Two different methods were used to assay the antifungal activity, namely: (a) disk diffusion test (Kirby–Bauer) and (b) deep diffusion test, by spread plate method (a) or by pour plate method (b).

The yeast pure strains were cultured on solid YPD media at 30 °C for 48 h to ensure viability and purity. The cell yeasts were then subcultured in a liquid YPD media for 14–18 h at 30 °C, and were used as a source of inoculum for each assay. The inocula (10^5^–10^7^CFU mL^−1^) were adjusted for each strain and each experiment. The antifungal sensitivity was assayed in a Müeller-Hinton agar (MHA) medium supplemented with glucose (2% *w*/*v*) and methylene blue (0.5 µg mL^−1^). The MHA was melted at 50 °C and then inoculated with 3 mL of yeast suspension prepared in a sterile saline solution (NaCl 0.9%) and adjusted between 0.1 and 0.4 Abs at 600 nm (10^5^–10^7^CFU mL^−1^). The plates were incubated at 30–37 °C for 24–48 h in the presence of different formulations.

#### 4.5.1. Disk Diffusion Test

Three AmB liquid formulations (reported in Table 2) were used. Sterile disks of high-grade cellulose (6 mm diameter) were embedded with 20 µL of each of the AmB formulations. Once the disks were dried, they were placed into yeast inoculated Müeller-Hinton plates. The plates were kept at 4 °C for 2 h, followed by 48 h at 37 °C for *Candida* spp. and 30 °C for *S. cerevisiae*. Finally, the diameter of the growth inhibition halos was measured. Each experiment was repeated three times.

#### 4.5.2. Deep Diffusion Test

Cylindrical holes of 7 mm diameter and 4 mm height with an empty volume of 0.15 mL were made into the yeast inoculated Müeller-Hinton plates. Then, 0.1 mL of each of the semisolid AmB A and AGCD formulations were placed into the holes in the agar plates. The incubations and the diameter of the growth inhibition halos were evaluated in the same way as the reported disk diffusion method.

### 4.6. Statistic Data Treatment

The experiments were performed at least in triplicate. Statistical significance was studied through Student’s t-test and ANOVA (Microsoft Office Professional Plus, Excel, 2016 (Microsoft, Redmond, WA, USA)). The mean results with the 95% confidence intervals are shown in the figures.

## 5. Conclusions

It can be concluded that AmB solubilization by γ-cyclodextrins enhances both skin accumulation and antifungal in vitro activity in comparison to AmB ointment.

## Figures and Tables

**Table 1:** Composition (%, *w*/*w*) of the semisolid formulations. Key: A semisolid AmB simple reference formulation, ADMSO is an AmB reference formulation with DMSO, and AGCD is an AmB formulation with γ-cyclodextrins.

Components	A	ADMSO	AGCD
AmB	0.125	0.125	0.125
Orabase^® 1^	99.875	87.375	87.375
DMSO	-	12.5	-
γ-cyclodextrin	-	-	12.5

^1^ Hydrophobic marketed standard base, for which main component is mineral oil.

**Table 2:** Composition (%, *w*/*w*) of the liquid formulations. Key: Suspension A is a liquid AmB simple reference formulation, ADMSO is an AmB solution reference formulation with DMSO, and AGCD is an AmB solution formulation with γ-cyclodextrins.

Components	Suspension A	Solution ADMSO	Solution AGCD
AmB	0.125	0.125	0.125
Purified water	99.875	-	87.375
DMSO	-	99.875	-
γ-cyclodextrin	-	-	12.5
